# COVID-19 hospitalizations and deaths averted under an accelerated vaccination program in northeastern and southern regions of the USA

**DOI:** 10.1016/j.lana.2021.100147

**Published:** 2021-12-29

**Authors:** Thomas N. Vilches, Pratha Sah, Seyed M. Moghadas, Affan Shoukat, Meagan C. Fitzpatrick, Peter J. Hotez, Eric C. Schneider, Alison P. Galvani

**Affiliations:** a Center for Infectious Disease Modeling and Analysis (CIDMA), Yale School of Public Health, New Haven, Connecticut, USA; b Agent-Based Modelling Laboratory, York University, Toronto, Ontario, Canada; c Center for Vaccine Development and Global Health, University of Maryland School of Medicine, Baltimore, Maryland, USA; d National School of Tropical Medicine, Baylor College of Medicine, Houston, USA; e The Commonwealth Fund, 1 East 75th Street, New York, NY 10021 USA

## Abstract

**Background:**

The fourth wave of COVID-19 pandemic peaked in the US at 160,000 daily cases, concentrated primarily in southern states. As the Delta variant has continued to spread, we evaluated the impact of accelerated vaccination on reducing hospitalization and deaths across northeastern and southern regions of the US census divisions.

**Methods:**

We used an age-stratified agent-based model of COVID-19 to simulate outbreaks in all states within two U.S. regions. The model was calibrated using reported incidence in each state from October 1, 2020 to August 31, 2021, and parameterized with characteristics of the circulating SARS-CoV-2 variants and state-specific daily vaccination rate. We then projected the number of infections, hospitalizations, and deaths that would be averted between September 2021 and the end of March 2022 if the states increased their daily vaccination rate by 20 or 50% compared to maintaining the status quo pace observed during August 2021.

**Findings:**

A 50% increase in daily vaccine doses administered to previously unvaccinated individuals is projected to prevent a total of 30,727 hospitalizations and 11,937 deaths in the two regions between September 2021 and the end of March 2022. Southern states were projected to have a higher weighted average number of hospitalizations averted (18.8) and lives saved (8.3) per 100,000 population, compared to the weighted average of hospitalizations (12.4) and deaths (2.7) averted in northeastern states. On a per capita basis, a 50% increase in daily vaccinations is expected to avert the most hospitalizations in Kentucky (56.7 hospitalizations per 100,000 averted with 95% CrI: 45.56 - 69.9) and prevent the most deaths in Mississippi, (22.1 deaths per 100,000 population prevented with 95% CrI: 18.0 - 26.9).

**Interpretation:**

Accelerating progress to population-level immunity by raising the daily pace of vaccination would prevent substantial hospitalizations and deaths in the US, even in those states that have passed a Delta-driven peak in infections.

**Funding:**

This study was supported by The Commonwealth Fund. SMM acknowledges the support from the Canadian Institutes of Health Research [OV4 – 170643, COVID-19 Rapid Research] and the Natural Sciences and Engineering Research Council of Canada, Emerging Infectious Disease Modelling, MfPH grant. MCF acknowledges support from the National Institutes of Health (5 K01 AI141576).

**Research in context**

**Evidence before this study**

The COVID-19 vaccination campaign in the US has been highly effective in reducing disease burden. However, despite the spike in hospitalizations and deaths due to the ongoing Delta variant wave, the pace of vaccine administration in the US slowed during summer to fewer than 700,000 doses per day. The impact of increasing vaccination rates in US states has not been evaluated.

**Added value of this study**

This study quantifies the population-level impact of accelerating vaccination rates in northeastern and southern states of the US. Using a multi-variant agent-based model, calibrated to state-specific data, we simulated COVID-19 transmission under status quo and compared the outcomes with scenarios in which daily vaccination rates increased by either 20 or 50%. We estimated that accelerating vaccination can still avert substantial COVID-19 outcomes of hospitalizations and deaths, even in states which have already experienced high levels of infection during the Delta wave. The benefits are largest for states with relatively lower vaccination coverage.

**Implications of all the available evidence**

A 50% increase in daily vaccination rate would avert substantial hospitalizations and deaths from the Delta variant. With the ongoing threat that new, highly-transmissible or immune-evading variants will emerge, our results underscore the need to accelerate the rise of population immunity through vaccination and not the natural infection that comes with the tragic costs of hospitalizations and loss of human lives.

Alt-text: Unlabelled box

## Introduction

The Delta variant of the SARS-CoV-2 virus, with an estimated uncontrolled reproduction number of 5.08,^1^ drove daily COVID-19 deaths in the United States (US) to over 2,000. At the peak of the Delta surge in August 2021, more than 100,000 people were hospitalized with COVID-19, the highest since the peak of the pandemic in January 2021 when most Americans were not yet eligible for vaccination. The surge hit southern states especially hard. For example, by mid-August, the seven-day average of new cases per 100,000 population exceeded 50 in southern states of Louisiana, Georgia, Alabama, Texas and Florida, while per capita reported cases in northern states of Wisconsin, New York, New Hampshire, Pennsylvania and Michigan were under 24.^2^ Data from Institute of Health Metrics and Evaluation indicate that, between May 1 and September 30, 2021, more than 100,000 mostly unvaccinated Americans in the southern US lost their lives from COVID-19 despite the availability of COVID-19 vaccines.^3^

Following a tragic loss of life and substantial costs of preventable hospitalizations and cases, the southern region COVID-19 outbreak has started to abate. However, the spread of the Delta variant has continued, especially in the Midwest, posing major challenges to pandemic control in the US over the next several weeks or months, with severe illness and death concentrated among the unvaccinated. A recent study estimated that the rates of infection and hospitalizations among unvaccinated individuals are 4.9 and 29.2 times higher, respectively, compared with those fully vaccinated,^4^ reflecting the vaccine effectiveness exceeding 50 and 90% in preventing infection and hospitalization, respectively.^5^

Vaccination is key to achieving control of the pandemic. Prevention of hospitalizations and deaths continues to depend on quickly vaccinating as many people as possible. Encouragingly, after months of stagnation, vaccine uptake increased throughout the US. In August 2021, over 14 million individuals received their first dose of vaccines in the US, which is 4 million doses more than the month of July. Yet the pace of vaccination prior to the start of the booster campaign in the US had slowed to fewer than 700,000 doses per day^6^ ー a pace that will leave millions vulnerable for many months to come. The percentage of the population vaccinated varies dramatically across states. Some states have fully vaccinated more than 70% of their entire populations while others have only recently crossed 40%.^7^

In this study, we projected the impact of accelerating COVID-19 vaccination in the northeastern and southern regions of the US census divisions using a multi-variant transmission dynamic model. By fitting the model to incidence data reported for each states and District of Columbia in these regions, we compared the expected hospitalizations and deaths under the scenarios of: (i) sustaining the rate of vaccination as the average of daily administered doses in August 2021 (status quo), and (ii) accelerating daily vaccination rate of status quo by 20% or 50% from the beginning of September 2021 (counterfactuals). For this comparison, we considered the states of Connecticut, Massachusetts, Maine, New Hampshire, New Jersey, New York, Pennsylvania, Rhode Island and Vermont in the northeastern region. The southern region includes Alabama, Arkansas, Delaware, District of Columbia, Florida, Georgia, Kentucky, Louisiana, Maryland, Mississippi, North Carolina, Oklahoma, South Carolina, Tennessee, Texas, Virginia and West Virginia.^8^

## Materials and methods

We expanded our age-stratified, agent-based model (ABM) of COVID-19 to simulate the spread of SARS-CoV-2 variants, including Alpha, Gamma, Delta, and Iota variants in addition to the original Wuhan-1 strain (Figure S1).^9^, ^10^, ^11^ The basic structure of the ABM includes a finite collection of agents that represent individuals living in a population, and a virtual environment in which agents interact.^12^ Agents are assigned specific attributes (e.g., age, comorbidities) and are characterized by time-dependent epidemiological statuses. We implemented a sampling process for disease specific parameters from their relevant distributions for each individual, which accounts for parameter variations.

The model was parameterized with the population demographics of the states studied here, disease characteristics and estimates of variant transmissibility, as well as age-specific risks of severe health outcomes due to COVID-19 (Appendix).^13^, ^14^, ^15^, ^16^, ^17^, ^18^, ^19^ We retrieved state-specific data on daily vaccine doses administered from the Centers for Disease Control and Prevention.^20^^,^^21^ Vaccine effectiveness against infection, symptomatic, and severe disease were derived from published studies (Appendix, Table S3), accounting for different vaccine types, each variant, and timelines for generation of immunity after the first and second doses (Appendix, Table S3). We calibrated the model to reported incidence in each state between October 1, 2020 and August 31, 2021 to determine the per contact transmission probability, and simulated the impact of vaccination from September 1, 2021 to the end of March 2022 (Figure S4). Variants of SARS-CoV-2 virus were introduced into the disease transmission dynamics of each state according to timelines reported by a tracking database of the lineage prevalence of COVID-19 variants in the US.^22^ Through stochastic Monte-Carlo simulations, we projected infections, hospitalizations, and deaths, and calculated mean and 95% credible intervals for outcomes averted using a bias-corrected and accelerated bootstrap method with 500 replications. Detailed description of the model, its parameterization, and implementation are provided in the Appendix. The simulation code is available at: https://github.com/thomasvilches/multiple_strains/tree/north_south_states.

## Role of the funding source

Dr. Schneider is an employee of the funding organization and participated in the formulation of research questions, interpretation of results, and review of the manuscript, but not the conduct, data collection, or analysis. The Commonwealth Fund did not play any role in the decision to submit the manuscript for publication.

## Results

We projected infections, hospitalizations, and deaths under the scenario of status quo and two counterfactuals for accelerated vaccination. Under the status quo scenario, the average coverage of two-dose vaccination without a booster dose was projected to reach 77% and 86% in southern and northeastern states, respectively, by the end March 2022. With accelerated vaccination, the corresponding coverages would rise to 81% and 87%, if daily vaccination rates were increased by 20%, and 83% and 87% with a 50% increase in daily vaccination rates.

**Figure 1:**
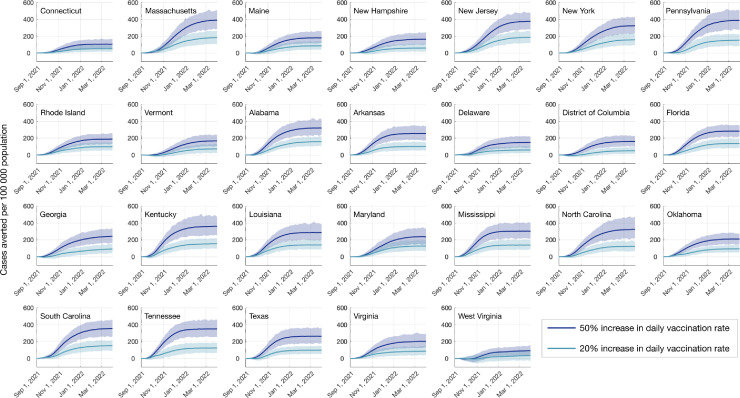
Cumulative COVID-19 cases averted with 50% and 20% increases in daily vaccination rate from the beginning of September 2021 to the end of March 2022, compared to maintaining the status quo pace observed during August 2021.

**Figure 2:**
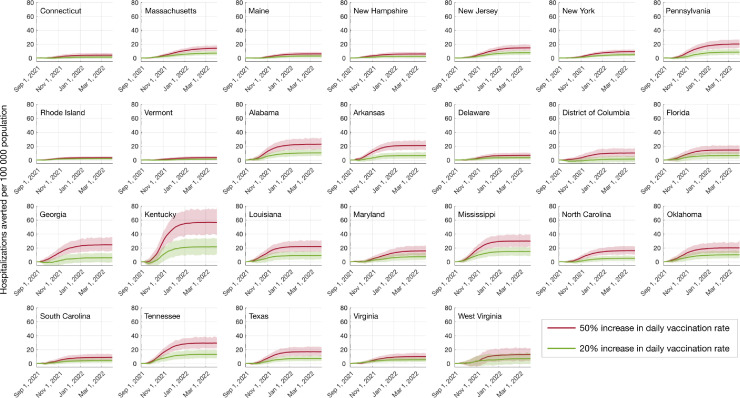
Cumulative COVID-19 hospitalizations averted with 50% and 20% increases in daily vaccination rate from the beginning of September 2021 to the end of March 2022, compared to maintaining the status quo pace observed during August 2021.

**Figure 3:**
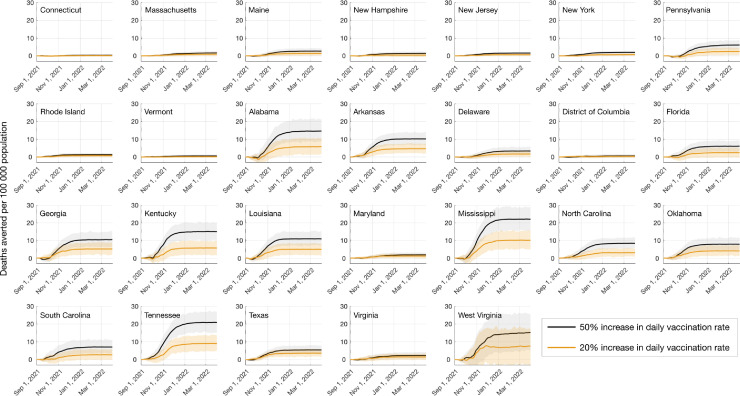
Cumulative COVID-19 deaths averted with 50 and 20% increases in daily vaccination rate from the beginning of September 2021 to the end of March 2022, compared to maintaining the status quo pace observed during August 2021.

**Figure 4:**
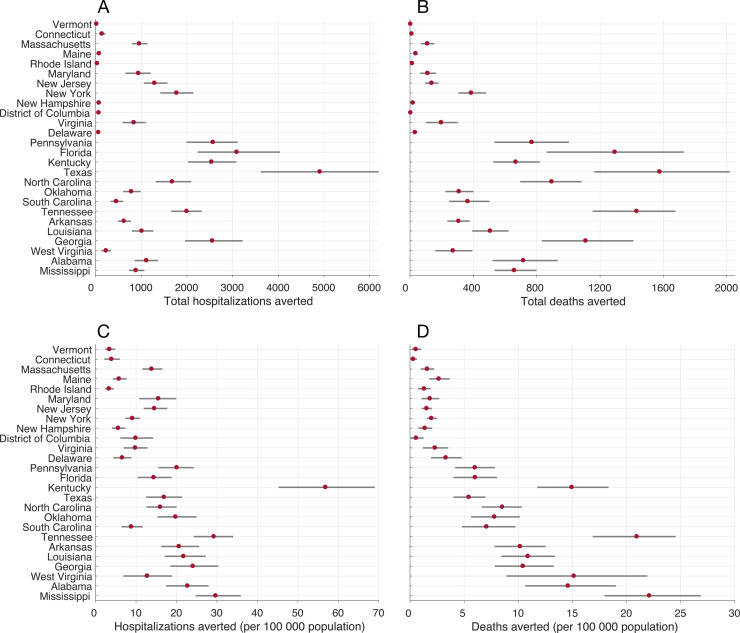
Projected overall (A,B) and per 100,000 population (C,D) hospitalizations and deaths averted by increasing the daily vaccination rate in each state by 50% from the average in August 2021. The estimates are cumulative from September 1, 2021 to March 31, 2022. States are ordered by their coverage of two-dose (full) vaccination on September 1, 2021.

Across all states in the two regions, a 50% increase in daily vaccination rates was projected to prevent 30,727 hospitalizations and 11,937 deaths (Figures 1–4) from the beginning of September 2021 to the end of March 2022. The average number of hospitalizations averted ranges from 21 (95% CrI: 14 - 29) in Vermont to 4,842 (95% CrI: 3,735 - 6,020) in Texas. The average number of deaths averted ranges from 4 (95% CrI: 1 - 7) to 1,554 (95% CrI: 1,088 - 2074) in the same two states (Figure 4A).

We also calculated the number of hospitalizations and deaths averted per capita under a 50% increase in daily rate of vaccination. Southern states were projected to have a higher weighted average number of hospitalizations averted (18.8) and lives saved (8.3) per 100,000 population, compared to the weighted average of hospitalizations (12.4) and deaths (2.7) averted in northeastern states. Accelerated vaccination would have the highest per capita impact in Kentucky, averting 56.7 (95% CrI: 45.6 - 69.9) hospitalizations, and in Mississippi, preventing 22.1 (95% CrI: 18.0 - 26.9) deaths per 100,000 population. Despite the severe summer Delta-wave in the south, accelerated vaccination would still have comparable or higher per capita impact in southern states compared to the northern states evaluated (Figure 4C, D). The lowest per capita impacts would be in Rhode Island and Connecticut on hospitalizations and deaths, respectively.

**Figure 5:**
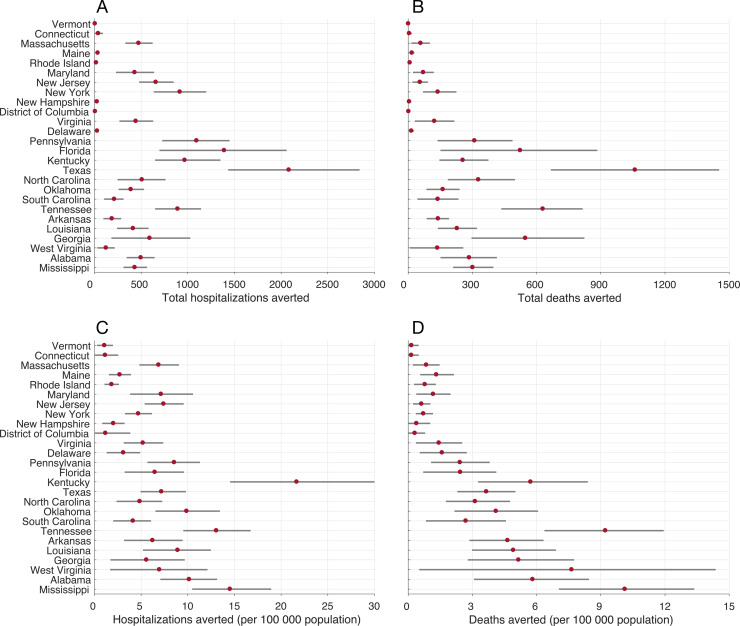
Projected overall (A,B) and per 100,000 population (C,D) hospitalizations and deaths averted by increasing the daily vaccination rate in each state by 20% from the average in August 2021. The estimates are cumulative from September 1, 2021 to March 31, 2022. States are ordered by their coverage of two-dose (full) vaccination on September 1, 2021.

With a lower increase in daily vaccination rates at 20%, we projected that additional 12,783 hospitalizations and 5,504 deaths would be averted across all states during the study period compared to the status quo (Figure 5). In the southern region, a weighted average of 7.6 hospitalizations and 3.9 deaths were projected to be averted, compared to the corresponding weighted averages of 5.8 and 1.1 in northeastern states.

## Discussion

Vaccination has dramatically reduced the toll of the COVID-19 pandemic.^23^, ^24^, ^25^, ^26^ Our results demonstrate that increasing vaccination rates can further reduce hospitalizations and save additional lives during the ongoing pandemic. Specifically, accelerating vaccination by 50% would avert 4.2 times more hospitalizations and save 9.3 times more lives per 100,000 population in the southern states compared to the northeastern states, even as a substantial late summer wave of COVID-19 in the south began to abate. While improved vaccine coverage can benefit all states, our results suggest that the benefits are larger in states with a lower vaccination coverage at baseline. For instance, southern states had an average coverage of 46.8% for full vaccination on September 1, 2021, compared to 63% in the northeastern states. Accelerating vaccination can bring states in both regions to comparable coverage by March 31, in part because the pace of vaccination was already somewhat higher in southern states during August 2021. However, accelerated vaccination remains beneficial even in the northeastern states, preventing 6,925 hospitalizations and 1,488 deaths between the start of September 2021 and the end of March 2022.

Our model incorporates current estimates of vaccine effectiveness against circulating variants and assumes that the Delta viral variant of SARS-CoV-2 will dominate for the next several months. However, continuous viral evolution, especially in settings with low vaccination coverage, could produce more transmissible or immune-evading variants. Such variants could reduce vaccination impact. In the absence of the underlying distributions, some model parameters were simplified to be constant based on the available estimates, including mean vaccine efficacies, the relative transmissibilities of SARS-COV-2 variants, and the risk of outcomes (e.g., hospitalizations or deaths) due to infection with different variants. We did not consider waning of naturally-acquired or vaccine-induced immunity within the study timeframe. Contact patterns of individuals were age dependent and sampled from empirically derived distributions without consideration of the location of occurrence (e.g., within households, workplaces, or schools) or the type of agent interactions. This is not expected to have a significant effect on our population-based results, because the model calibration and fitting to incidence data in each state would modulate the average per-contact transmission probability. However, we note that the reported incidence is likely undercounting the true number of COVID-19 infections. Lastly, we did not explicitly include geographic variability in scale or effect of interventions; but contacts were adjusted following calibration to fit the model to the number of cases in each state, implicitly accounting for the effect of non-pharmaceutical measures.

Despite these limitations, our findings indicate that increasing vaccination rates, even in states that have already passed the peak of the Delta wave, could greatly mitigate severe illness and deaths. With the expected seasonal rise of infections in northern Midwest, Mountain West, and northeastern states, accelerating vaccination is critical to avoid hospitalizations, strained healthcare, and significant loss of lives.

## Data availability

All data, parameters, and the computational codes used for the study are available at https://github.com/thomasvilches/multiple_strains/tree/north_south_states.

## Declaration of interests

None

## Appendix Supplementary materials

Image, application 1
