# Supplementary material for: COVID-19 hospitalizations and deaths averted under an accelerated vaccination program in northeastern and southern regions of the USA
Source: Lancet Reg Health Am. 2021 Dec 29;6:100147. doi: 10.1016/j.lana.2021.100147 (PMC8714489; doi:10.1016/j.lana.2021.100147)
Supplement: Supplementary file 1 [file mmc1.docx]

**Appendix**

# **COVID-19 hospitalizations and deaths averted under an accelerated vaccination program in northeastern and southern regions of the USA**

Thomas N. Vilches, PhD^1,2*^, Pratha Sah, PhD^1*^, Seyed M. Moghadas, PhD^2*^, Affan Shoukat, PhD^1^, Meagan C. Fitzpatrick, PhD^3^, Peter J Hotez, MD, PhD^4^, Eric C. Schneider, MD^5^, Alison P. Galvani, PhD^1^

1 Center for Infectious Disease Modeling and Analysis (CIDMA), Yale School of Public Health, New Haven, Connecticut, USA

2 Agent-Based Modelling Laboratory, York University, Toronto, Ontario, Canada

3 Center for Vaccine Development and Global Health, University of Maryland School of Medicine, Baltimore, Maryland, USA

4 National School of Tropical Medicine, Baylor College of Medicine, Houston, USA

5 The Commonwealth Fund, 1 East 75th Street, New York, NY 10021 USA

*Authors contributed equally

Corresponding Author: Alison P. Galvani. Center for Infectious Disease Modeling and Analysis (CIDMA), Yale School of Public Health, New Haven, Connecticut, USA (alison.galvani@yale.edu).

**Model structure**

We expanded a previous agent-based model of COVID-19 transmission[^1^](https://paperpile.com/c/rrZX91/BQbSv) to evaluate the effect of an accelerated vaccination campaign on reduction of hospitalizations and deaths in 25 states and the District of Columbia in northeast and southern regions of the US determined by the census bureau division.[^2^](https://paperpile.com/c/rrZX91/v2FM) In addition to encapsulating the demographics of each state,[^3^](https://paperpile.com/c/rrZX91/LasK8) the model included variants of Alpha, Gamma, Iota, Delta, and the original Wuhan-I strain. Timelines for the introduction of each variant in the transmission dynamics of each state were derived and implemented in the model based on the lineage prevalence of COVID-19 variants in the US provided by an open-source tracking database[^4^](https://paperpile.com/c/rrZX91/KTz0).

We implemented the natural history of disease by representing the epidemiological statuses of individuals as susceptible; latently infected (not yet infectious); asymptomatic (and infectious); pre-symptomatic (and infectious); symptomatic (and infectious) with either mild or severe illness; recovered; and dead (Figure S1). The model population was stratified into six age groups of 0 to 4, 5 to 19, 20 to 49, 50 to 64, 65 to 79, and 80+ years, accounting for age-specific risk of hospitalization and death.[^3^](https://paperpile.com/c/rrZX91/LasK8) Daily contacts between individuals were sampled from a negative-binomial distribution parameterized (Table S1) using empirical data on pre-pandemic and pandemic-era interactions.[^5,6^](https://paperpile.com/c/rrZX91/jqABC+FBkXB)

**Table S1.** Mixing patterns and the daily number of contacts derived from empirical observations.[^5,6^](https://paperpile.com/c/rrZX91/jqABC+FBkXB) Daily numbers of contacts were sampled from negative binomial distributions for different scenarios.

| **Age group** | **Proportion of contacts between age groups** | | | | | **No. of daily contacts without self-isolation**  **Mean (SD)** | **No. of daily contacts for self-isolated individuals**  **Mean (SD)** |
| --- | --- | --- | --- | --- | --- | --- | --- |
|  | **0-4** | **5-19** | **20-49** | **50-65** | **65+** |  |  |
| 0-4 | 0.2287 | 0.1839 | 0.4219 | 0.1116 | 0.0539 | 10.21 (7.65) | 2.86 (2.14) |
| 5-19 | 0.0276 | 0.5964 | 0.2878 | 0.0591 | 0.0291 | 16.793 (11.7201) | 4.70 (3.28) |
| 20-49 | 0.0376 | 0.1454 | 0.6253 | 0.1423 | 0.0494 | 13.795 (10.5045) | 3.86 (2.95) |
| 50-65 | 0.0242 | 0.1094 | 0.4867 | 0.2723 | 0.1074 | 11.2669 (9.5935) | 3.15 (2.66) |
| 65+ | 0.0207 | 0.1083 | 0.4071 | 0.2193 | 0.2446 | 8.0027 (6.9638) | 2.24 (1.95) |

**Transmissibility**

We calibrated the model to determine the per-contact transmission probability of the original Wuhan-I strain during the pre-symptomatic stage of the disease by fitting the model to case incidence data per 100,000 population in each state from October 1, 2020, to August 31, 2021.[^7^](https://paperpile.com/c/rrZX91/XVDhq) The transmissibility of asymptomatic, mild symptomatic, and severe symptomatic states was set to 26%, 44%, and 89% relative to the pre-symptomatic stage.[^8–10^](https://paperpile.com/c/rrZX91/UOJnS+MRhLI+GdRB6) The transmissibilities of Alpha, Gamma, Iota, were assumed to be 50%, 60%, 35% higher than the original strain, and the transmissibility of Delta was assumed to be 30% higher than Alpha.[^11–16^](https://paperpile.com/c/rrZX91/DDizF+wZeIt+Tw3Mw+TCTQ4+EJZx9+bJvAb)

We calibrated our model to incidence data rather than hospitalizations or death data because the latter datasets are likely skewed towards older age groups and those at high-risk of severe outcomes (i.e., those with comorbid conditions). Additionally, fitting to incidence data allowed us to determine the per-contact transmission probability in each state. Using datasets of severe outcomes for fitting may not reflect the transmissibility or the effect of interventions in the population. The fitting to incidence provided trends of hospitalizations and deaths which matched those observed in reported data.

**Disease dynamics**

The incubation period was sampled for each infected individual from a log-normal distribution with a mean of 5.2 days.[^17^](https://paperpile.com/c/rrZX91/z4TKc) An age-dependent proportion of infected individuals progressed to a pre-symptomatic stage[^18^](https://paperpile.com/c/rrZX91/Udshg) with a mean duration of 2.3 days, sampled from a Gamma distribution.[^9,19^](https://paperpile.com/c/rrZX91/YftGE+MRhLI) Pre-symptomatic cases developed symptomatic disease with an average infectious period of 3.2 days, which was also sampled from a Gamma distribution.[^20,21^](https://paperpile.com/c/rrZX91/aQQrw+4rkID) Infected individuals who remained asymptomatic had an average infectious period of 5 days sampled from a Gamma distribution.[^20,21^](https://paperpile.com/c/rrZX91/aQQrw+4rkID)

**Immune evasion**

We assumed that the Gamma and Delta variants evade naturally-acquired immunity by an average of 21% (95% CI: 11-36%).[^22,23^](https://paperpile.com/c/rrZX91/TszrC+2wRyL) This evasion rate [^24–27^](https://paperpile.com/c/rrZX91/JxMAJ+AMpwd+6yydC+GNmG7) was implemented as a reduction of immune protection for individuals recovered from the original strain or the Alpha/Iota variants. We further assumed that recovery from infection due to the Gamma or Delta variant provides protection against all variants in the model, preventing reinfection for the duration of the study.

**Infection outcomes**

All asymptomatic and mild symptomatic cases were assumed to recover from infection without hospitalization or death. Severely ill cases were hospitalized within 2-5 days of symptom onset[^28,29^](https://paperpile.com/c/rrZX91/jVZ3r+IVVyo) and therefore had no contribution to further spread of infection. Symptomatic cases who were not hospitalized self-isolated within 24 hours of symptom onset, reducing their number of daily contacts by an average of 74% (Table S1). Intensive care unit (ICU) and non-ICU hospitalization rates were parameterized (Table S2) by clinical and epidemiological data.[^30–32^](https://paperpile.com/c/rrZX91/ZRsJm+d04oO+v3zl8) The risk of hospitalization with the Delta variant was assumed to be 2.26 times higher than that due to infection with Alpha.[^33^](https://paperpile.com/c/rrZX91/RvKRP) Infection with Alpha and Delta variants was associated with 30% higher risk of death.[^12,13^](https://paperpile.com/c/rrZX91/wZeIt+Tw3Mw)

**Table S2.** Model parameters associated with hospitalization of severe cases.

| Proportion of severe cases hospitalized with one or more comorbidities | | 100% | [^30–32^](https://paperpile.com/c/rrZX91/ZRsJm+d04oO+v3zl8) |
| --- | --- | --- | --- |
|  | Non-ICU | 60.4% |  |
|  | ICU | 39.6% |  |
| Proportion of severe cases hospitalized without any comorbidities | | 10.8% | [^30–32^](https://paperpile.com/c/rrZX91/ZRsJm+d04oO+v3zl8) |
|  | Non-ICU | 75% |  |
|  | ICU | 25% |  |
| Length of non-ICU stay (days) | | Gamma(shape: 4.5, scale: 2.75) | Derived from  [^34,35^](https://paperpile.com/c/rrZX91/V1LbM+EG0i6) |
| Length of ICU stay  (days) | | Gamma(shape: 4.5, scale: 2.75) + 2 | Derived from  [^34,35^](https://paperpile.com/c/rrZX91/V1LbM+EG0i6) |

**Vaccination**

Vaccination was implemented as a two-dose strategy from December 12, 2020, with a sequential prioritization of (i) healthcare workers (5% of the total population)[^36^](https://paperpile.com/c/rrZX91/LD8Pv), adults with comorbidities, and those aged 65 and older; and (ii) other individuals aged 16-64 [^37,38^](https://paperpile.com/c/rrZX91/8kRYR+et2ZS). We parameterized the model with the daily number of vaccine doses administered in different age groups resembling the trends at the national level (Figure S2).[^39,40^](https://paperpile.com/c/rrZX91/RJgvO+dgtXF) The minimum age-eligibility for vaccination was 16 years before May 13, 2021 after which children aged 12 to 15 years became eligible for vaccination. We did not include vaccination of children under the age of 12 in the model, although their vaccination with Pfizer-BioNTech vaccines has recently been approved[^41^](https://paperpile.com/c/rrZX91/sQ1n).

The time interval between first and second doses were specified to be 21 and 28 days for Pfizer-BioNTech and Moderna vaccines, respectively. [^42^](https://paperpile.com/c/rrZX91/XVlPT)^,^[^43^](https://paperpile.com/c/rrZX91/gWdhH) The model was parameterized with published estimates of the mean vaccine efficacy following each dose of vaccines (Table S3) against infection (i.e., reduction of transmission probability), symptomatic disease (i.e., reduction in the probability of developing symptomatic disease), and severe disease (i.e., reduction of severe illness if symptomatic disease occurred). [^44–47^](https://paperpile.com/c/rrZX91/dJ8bP+vp9BL+z9xUy+qStuy)

**Table S3.** Estimated vaccine efficacies (%) and their 95% confidence intervals from published studies.

| **Vaccine efficacy (%)** | **Weeks after the first dose** | | **Weeks after the second dose** | | **Reference** |
| --- | --- | --- | --- | --- | --- |
| Original/Iota strain | 1-2 | 3 | 1-2 | >2 | [^43,46,48–51^](https://paperpile.com/c/rrZX91/gWdhH+YxwyK+Y2mrc+4hevB+r6n0k+z9xUy) |
| Infection | None | 46 (40, 51) | 60 (53, 66) | 86.1 (82.4, 89.1) |  |
| Symptomatic disease | None | 57 (50, 63) | 66 (57, 73) | 94 (87, 98) |  |
| Severe disease | None | 62 (39, 80) | 80 (59, 94) | 92 (75, 100) |  |
| Alpha variant | 1-2 | 3 | 1-2 | >2 | [^47,52,53^](https://paperpile.com/c/rrZX91/qStuy+2i98o+exKSj) |
| Infection | None | 29.5 (22.9, 35.5) | 60 (53, 66) | 89.5 (85.9, 92.3) |  |
| Symptomatic disease | None | 53.6 (50, 63) | 62 (57, 73) | 93.7 (91.6, 95.3) |  |
| Severe disease | None | 54.1 (26.1, 71.9) | 80 (59, 94) | 94 (87, 98) |  |
| Gamma/Beta variant * | 1-2 | 3 | 1-2 | >2 | [^47,52^](https://paperpile.com/c/rrZX91/qStuy+2i98o) |
| Infection | None | 36.8 (32., 40.8) | 48 (42.4, 52) | 73.6 (70.4, 76) |  |
| Symptomatic disease | None | 33.2 (8.3, 51.4) | 66 (57, 73) | 94 (87, 98) |  |
| Severe disease | None | 34 (0, 50) | 68 (64, 75) | 97.4 (92.2, 99.5) |  |
| Delta variant | 1-2 | 3 | 1-2 | >2 | [^53–56^](https://paperpile.com/c/rrZX91/exKSj+JorjV+4TjXL+FM7HK) |
| Infection | None | 36.8 (32.0, 40.8) | 48 (42.4, 52) | 64 (57, 70) |  |
| Symptomatic disease | None | 33.5 (20.6, 44.3) | 62 (57, 73) | 88 (85.3, 90.1) |  |
| Severe disease | None | 34 (0, 50) | 68 (64, 75) | 80 (73–85) |  |

* Vaccine efficacy against the Gamma variant was assumed to be the same as those reported for Beta.

We considered vaccine distributions and the efficacy data of the Pfizer-BioNTech and Moderna vaccines. Combined, these two vaccines constitute ~96% of doses administered in the United States. A third single-dose vaccine developed by Johnson and Johnson is also authorized, which has a slightly lower efficacy compared to the Pfizer-BioNTech and Moderna vaccines.[^57,58^](https://paperpile.com/c/rrZX91/vmMM+qZtq) Since counterfactual scenarios were compared with the status quo (in which we also used Pfizer-BioNTech and Moderna vaccines), we do not expect lower efficacy of Johnson and Johnson vaccines in preventing infection, symptomatic and severe disease, to have a significant impact estimates regarding vaccination impact on reducing COVID-19 burden.

For the scenario of accelerated vaccination, we assumed that each state increased the average number of vaccine doses administered during August 2021 (Figure S1) by 20% or 50% from the beginning of September.


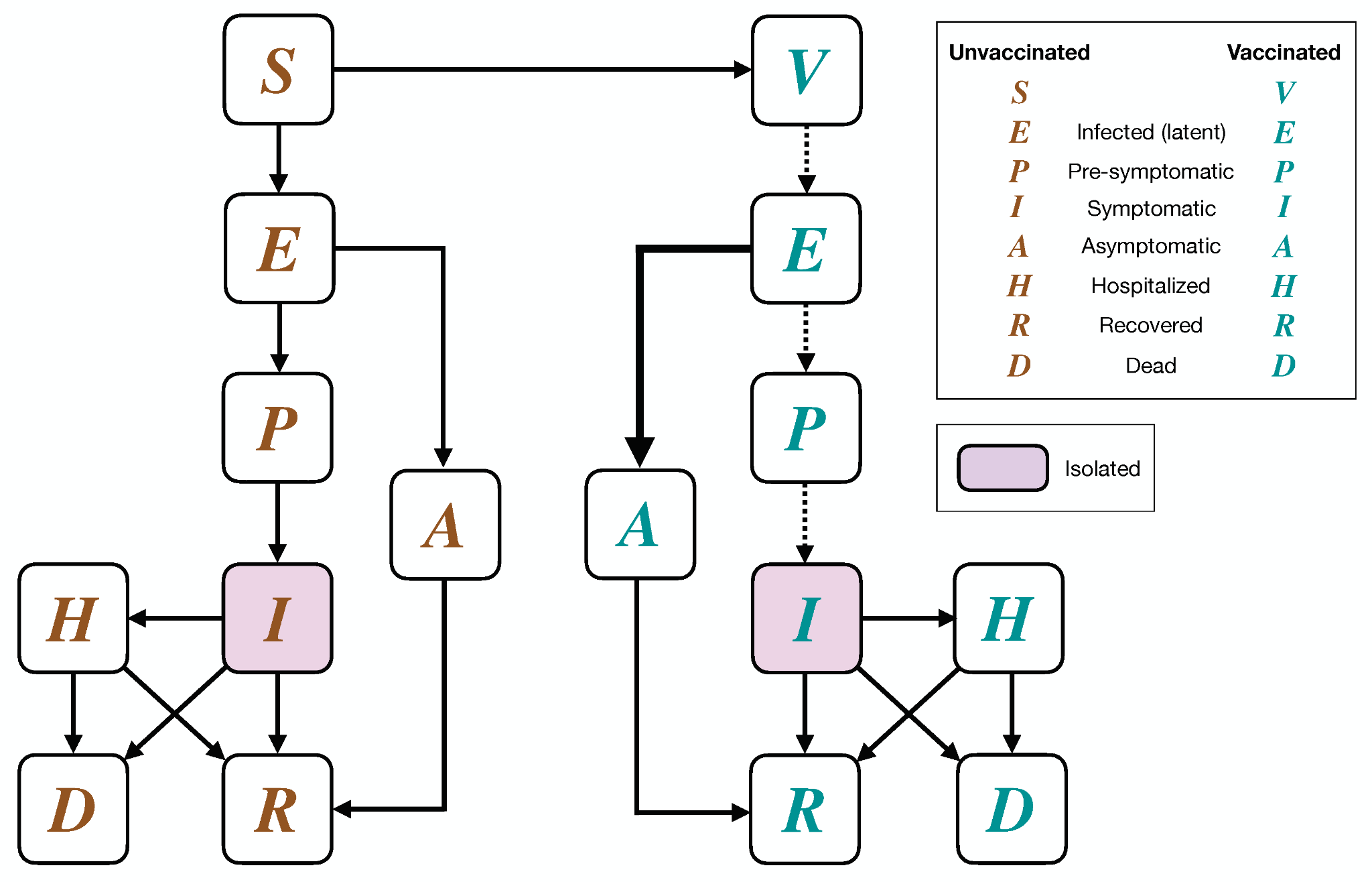


**Figure S1.** Schematic diagram of disease dynamics and vaccination in the model.

**
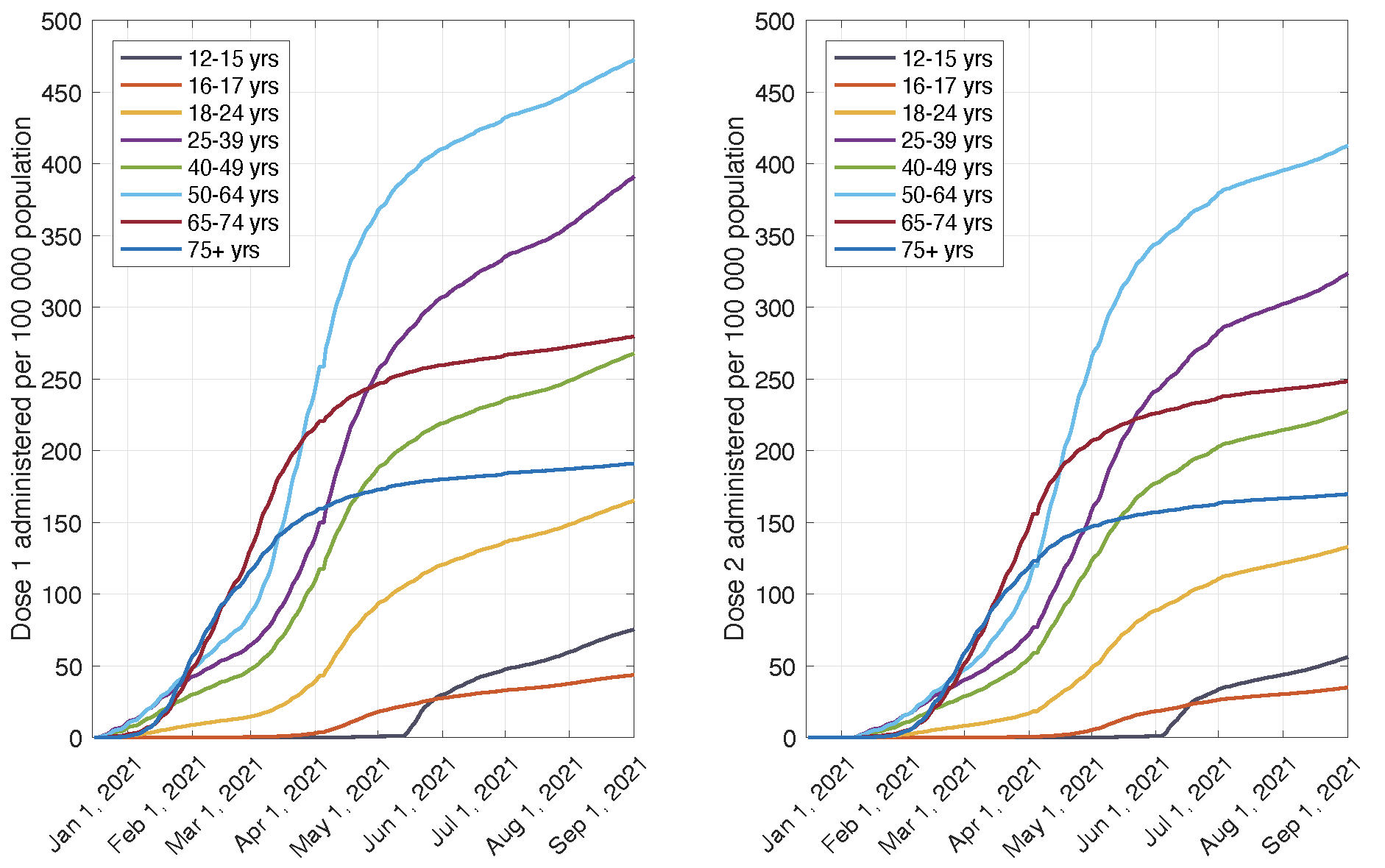
**

**Figure S2.** Age-specific temporal vaccination rates per 100,000 population obtained from CDC.[^59^](https://paperpile.com/c/rrZX91/FeBG)

**
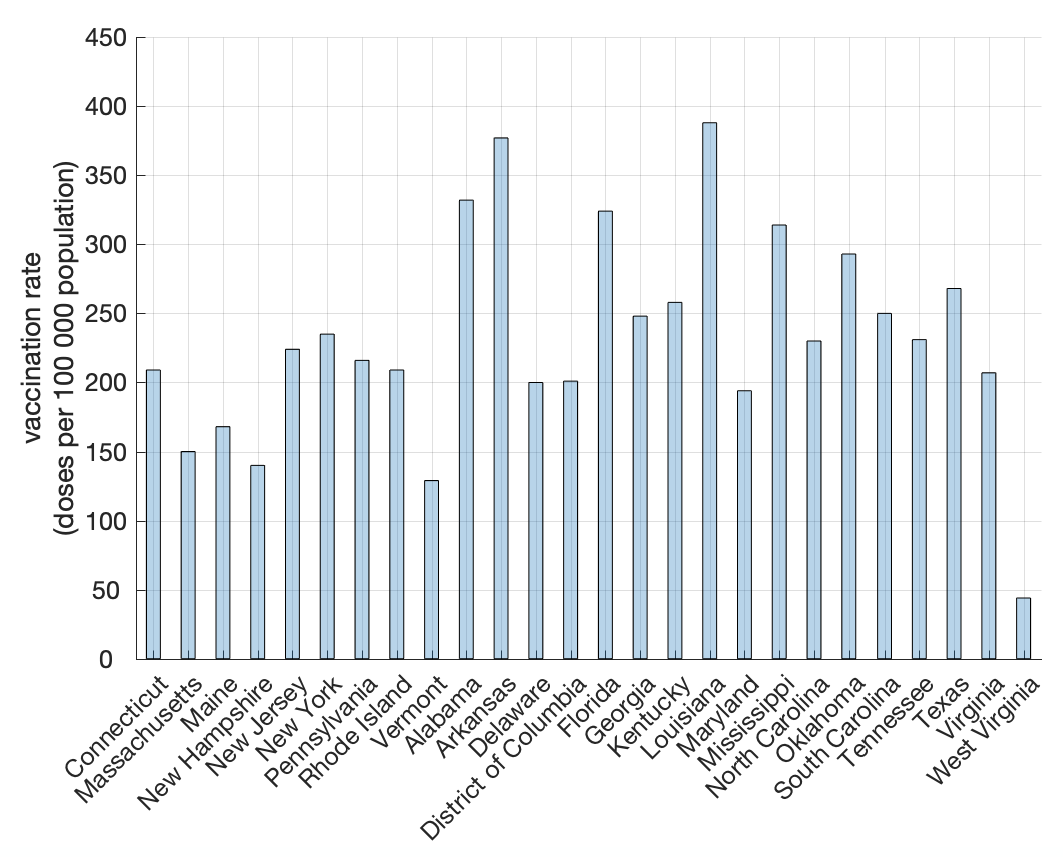
**

**Figure S3.** Average number of vaccine doses administered per 100,000 population during August 2021 in northeastern and southern states of the US.


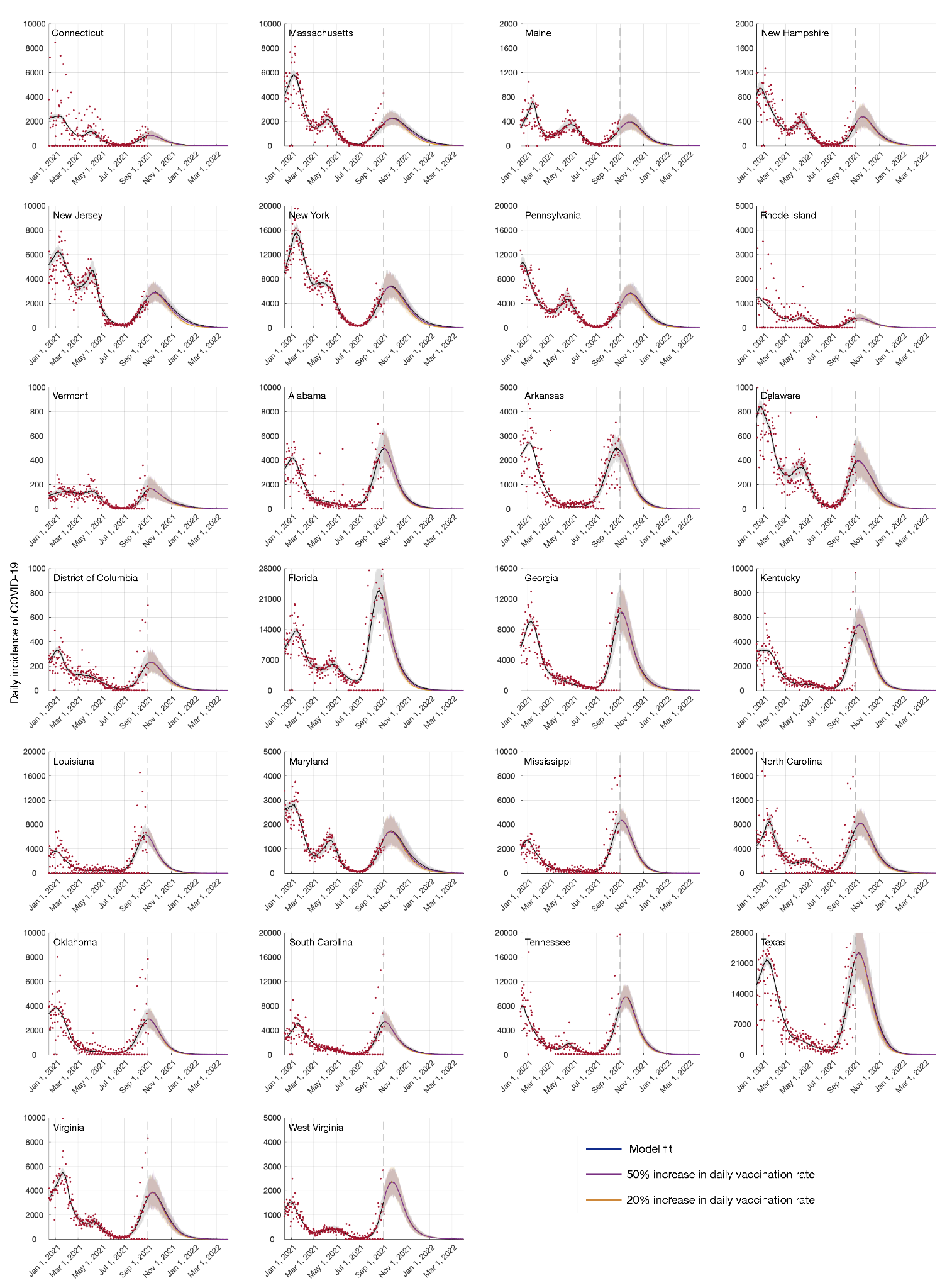


**Figure S4.** Model fit (black curves) to incidence data (red dots) and projected incidence in simulated scenarios of status quo and accelerated vaccination with 50% (orange curves) and 20% (purple curve) increase in daily vaccination rate from September 1, 2021 to March 31, 2022.

**Model implementation**

Model fitting was performed from October 1, 2020 to August 31, 2021 for each state with a population of 100,000 individuals, assuming that the viral landscape of SARS-CoV-2 will remain unchanged in the next several months. The model outcomes were scaled to the population size for each state using a bias-corrected and accelerated bootstrap method (with 500 replications). The pre-existing immunity in the population for each state was implemented at the start of simulations.[^60^](https://paperpile.com/c/rrZX91/LMiif) The model was simulated using daily time-steps from September 1, 2021 to March 31, 2022 to project the number of cases, hospitalizations, and deaths in two scenarios: (i) daily vaccination rate remains the same as 7-day average of the last week of August 2021 in each state (status quo); (ii) daily vaccination rate increased by either 20% or 50% (accelerated vaccination) compared to the status quo. We fixed the per-contact probability of transmission for the original strain that was determined in the calibration process for each state. During the simulations, the age-specific contact rates were adjusted for fitting the model by minimizing the error between temporal cumulative incidence derived from simulations and observed data, implicitly accounting for the change and effect of various non-pharmaceutical measures. For each state and scenario, simulations were averaged over 500 independent Monte-Carlo realizations, and 95% credible intervals derived. The model was implemented in Julia, and simulation codes are available at: https://github.com/thomasvilches/multiple_strains/tree/us_states.

**
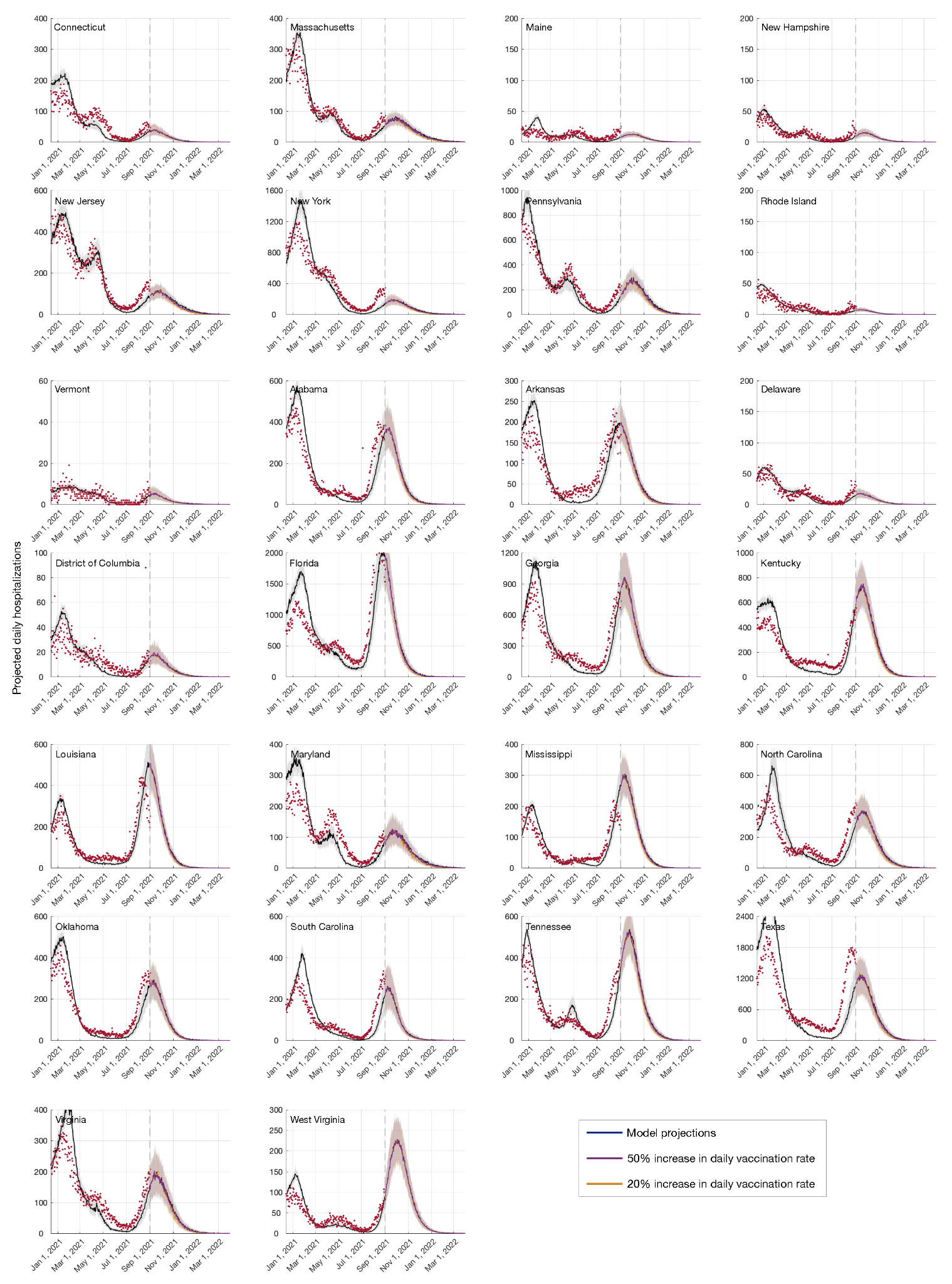
**

**Figure S5.** Model projections (black curves) of hospitalizations and simulated scenarios of status quo and accelerated vaccination with 50% (orange curves) and 20% (purple curve) increase in daily vaccination rate from September 1, 2021 to March 31, 2022. Red dots represent reported hospitalizations.


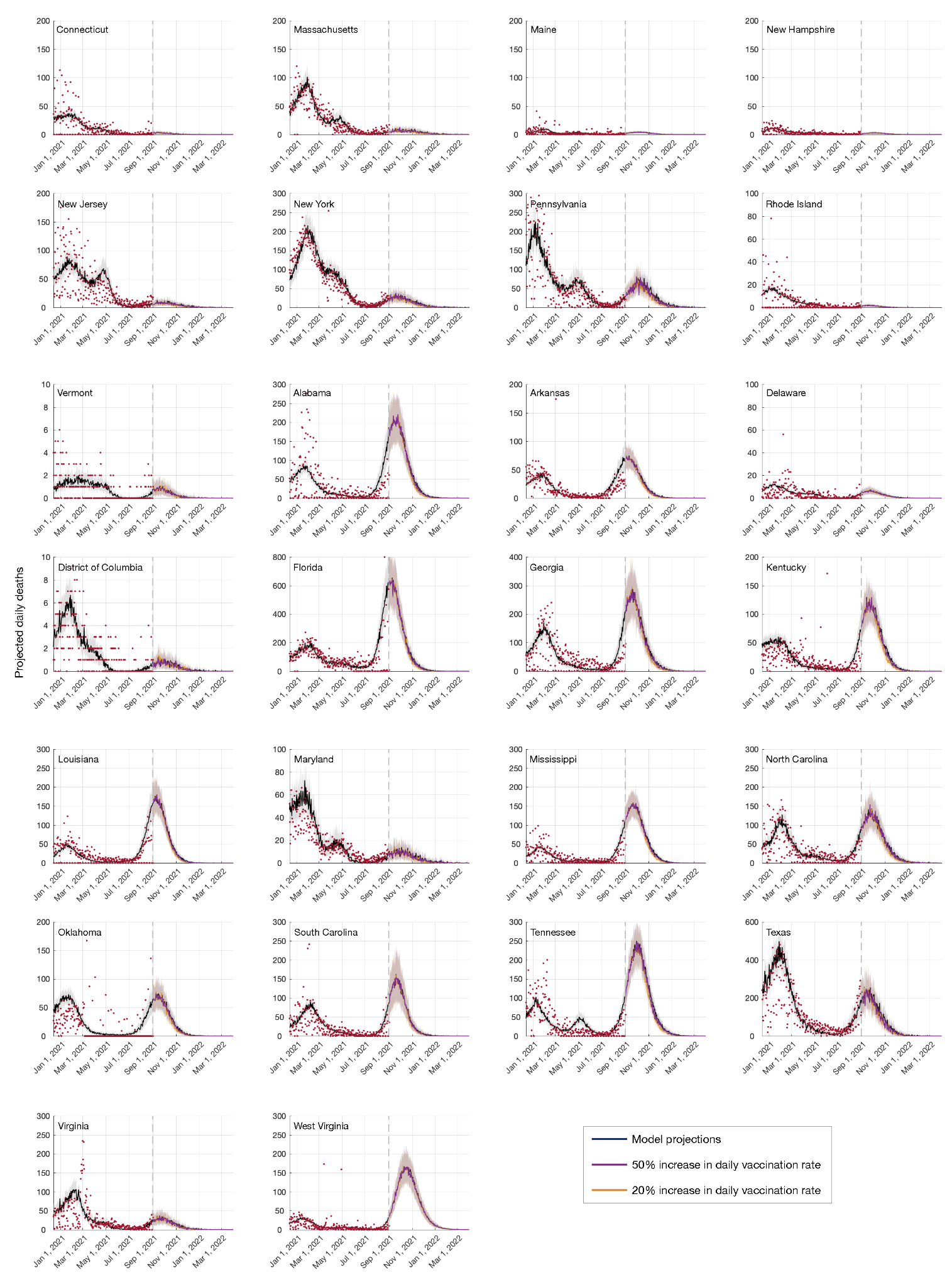
**Figure S6.** Model projections (black curves) of deaths and simulated scenarios of status quo and accelerated vaccination with 50% (orange curves) and 20% (purple curve) increase in daily vaccination rate from September 1, 2021 to March 31, 2022. Red dots represent reported hospitalizations.

**Reference**

1 [Moghadas SM, Sah P, Fitzpatrick MC, *et al.* COVID-19 deaths and hospitalizations averted by rapid vaccination rollout in the United States. *medRxiv* 2021; : 2021.07.07.21260156.](http://paperpile.com/b/rrZX91/BQbSv)

2 [US Census Bureau. 2010 Census Regions and Divisions of the United States.](http://paperpile.com/b/rrZX91/v2FM) <https://www.census.gov/geographies/reference-maps/2010/geo/2010-census-regions-and-divisions-of-the-united-states.html> [(accessed Nov 15, 2021).](http://paperpile.com/b/rrZX91/v2FM)

3 [US Census Bureau. State population by characteristics: 2010-2019.](http://paperpile.com/b/rrZX91/LasK8) <https://www.census.gov/data/tables/time-series/demo/popest/2010s-state-detail.html> [(accessed Aug 12, 2021).](http://paperpile.com/b/rrZX91/LasK8)

4 [outbreak.info.](http://paperpile.com/b/rrZX91/KTz0) <https://outbreak.info/location-reports?loc=USA> [(accessed Oct 4, 2021).](http://paperpile.com/b/rrZX91/KTz0)

5 [Mossong J, Hens N, Jit M, *et al.* Social contacts and mixing patterns relevant to the spread of infectious diseases. *PLoS Med* 2008; **5**: e74.](http://paperpile.com/b/rrZX91/jqABC)

6 [Jarvis CI, Van Zandvoort K, Gimma A, *et al.* Quantifying the impact of physical distance measures on the transmission of COVID-19 in the UK. *BMC Med* 2020; **18**: 124.](http://paperpile.com/b/rrZX91/FBkXB)

7 [The New York Times. Coronavirus (Covid-19) Data in the United States. Github, 2021](http://paperpile.com/b/rrZX91/XVDhq) <https://github.com/nytimes/covid-19-data> [(accessed May 1, 2021).](http://paperpile.com/b/rrZX91/XVDhq)

8 [Ferretti L, Wymant C, Kendall M, *et al.* Quantifying SARS-CoV-2 transmission suggests epidemic control with digital contact tracing. *Science* 2020; **368**. DOI:](http://paperpile.com/b/rrZX91/UOJnS)[10.1126/science.abb6936](http://dx.doi.org/10.1126/science.abb6936)[.](http://paperpile.com/b/rrZX91/UOJnS)

9 [Moghadas SM, Fitzpatrick MC, Sah P, *et al.* The implications of silent transmission for the control of COVID-19 outbreaks. *Proc Natl Acad Sci U S A* 2020; **117**: 17513–5.](http://paperpile.com/b/rrZX91/MRhLI)

10 [Sayampanathan AA, Heng CS, Pin PH, Pang J, Leong TY, Lee VJ. Infectivity of asymptomatic versus symptomatic COVID-19. *Lancet* 2021; **397**: 93–4.](http://paperpile.com/b/rrZX91/GdRB6)

11 [Davies NG, Abbott S, Barnard RC, *et al.* Estimated transmissibility and impact of SARS-CoV-2 lineage B.1.1.7 in England. *Science* 2021; **372**. DOI:](http://paperpile.com/b/rrZX91/DDizF)[10.1126/science.abg3055](http://dx.doi.org/10.1126/science.abg3055)[.](http://paperpile.com/b/rrZX91/DDizF)

12 [Davies NG, Jarvis CI, CMMID COVID-19 Working Group, *et al.* Increased mortality in community-tested cases of SARS-CoV-2 lineage B.1.1.7. *Nature* 2021; published online March 15. DOI:](http://paperpile.com/b/rrZX91/wZeIt)[10.1038/s41586-021-03426-1](http://dx.doi.org/10.1038/s41586-021-03426-1)[.](http://paperpile.com/b/rrZX91/wZeIt)

13 [Challen R, Brooks-Pollock E, Read JM, Dyson L, Tsaneva-Atanasova K, Danon L. Risk of mortality in patients infected with SARS-CoV-2 variant of concern 202012/1: matched cohort study. *BMJ* 2021; **372**: n579.](http://paperpile.com/b/rrZX91/Tw3Mw)

14 [Faria NR, Mellan TA, Whittaker C, *et al.* Genomics and epidemiology of the P.1 SARS-CoV-2 lineage in Manaus, Brazil. *Science* 2021; **372**: 815–21.](http://paperpile.com/b/rrZX91/TCTQ4)

15 [Annavajhala MK, Mohri H, Wang P, *et al.* A Novel and Expanding SARS-CoV-2 Variant, B.1.526, Identified in New York. *medRxiv* 2021; published online June 12. DOI:](http://paperpile.com/b/rrZX91/EJZx9)[10.1101/2021.02.23.21252259](http://dx.doi.org/10.1101/2021.02.23.21252259)[.](http://paperpile.com/b/rrZX91/EJZx9)

16 [Allen AH, Vusirikala A, Flannagan J, *et al.* Increased household transmission of COVID-19 cases associated with SARS-CoV-2 Variant of Concern B.1.617.2: a national case- control study.](http://paperpile.com/b/rrZX91/bJvAb) <https://khub.net/documents/135939561/405676950/Increased+Household+Transmission+of+COVID-19+Cases+-+national+case+study.pdf/7f7764fb-ecb0-da31-77b3-b1a8ef7be9aa> [(accessed July 1, 2021).](http://paperpile.com/b/rrZX91/bJvAb)

17 [Li Q, Guan X, Wu P, *et al.* Early Transmission Dynamics in Wuhan, China, of Novel Coronavirus-Infected Pneumonia. *N Engl J Med* 2020; **382**: 1199–207.](http://paperpile.com/b/rrZX91/z4TKc)

18 [Sah P, Fitzpatrick MC, Zimmer CF, *et al.* Asymptomatic SARS-CoV-2 infection: A systematic review and meta-analysis. *Proc Natl Acad Sci U S A* 2021; **118**. DOI:](http://paperpile.com/b/rrZX91/Udshg)[10.1073/pnas.2109229118](http://dx.doi.org/10.1073/pnas.2109229118)[.](http://paperpile.com/b/rrZX91/Udshg)

19 [He X, Lau EHY, Wu P, *et al.* Temporal dynamics in viral shedding and transmissibility of COVID-19. *Nat Med* 2020; **26**: 672–5.](http://paperpile.com/b/rrZX91/YftGE)

20 [Li R, Pei S, Chen B, *et al.* Substantial undocumented infection facilitates the rapid dissemination of novel coronavirus (SARS-CoV-2). *Science* 2020; **368**: 489–93.](http://paperpile.com/b/rrZX91/aQQrw)

21 [Gatto M, Bertuzzo E, Mari L, *et al.* Spread and dynamics of the COVID-19 epidemic in Italy: Effects of emergency containment measures. *Proc Natl Acad Sci U S A* 2020; **117**: 10484–91.](http://paperpile.com/b/rrZX91/4rkID)

22 [Centre for Mathematical Modelling of Infectious Diseases. Estimates of severity and transmissibility of novel SARS-CoV-2 variant 501Y.V2 in South Africa. Estimates of severity and transmissibility of novel SARS-CoV-2 variant 501Y.V2 in South Africa. 2021; published online Jan 11.](http://paperpile.com/b/rrZX91/TszrC) <https://cmmid.github.io/topics/covid19/sa-novel-variant.html> [(accessed May 2, 2021).](http://paperpile.com/b/rrZX91/TszrC)

23 [Jangra S, Ye C, Rathnasinghe R, *et al.* SARS-CoV-2 spike E484K mutation reduces antibody neutralisation. *Lancet Microbe* 2021; **2**: e283–4.](http://paperpile.com/b/rrZX91/2wRyL)

24 [Zhou D, Dejnirattisai W, Supasa P, *et al.* Evidence of escape of SARS-CoV-2 variant B.1.351 from natural and vaccine-induced sera. *Cell* 2021; **184**: 2348–61.e6.](http://paperpile.com/b/rrZX91/JxMAJ)

25 [Planas D, Bruel T, Grzelak L, *et al.* Sensitivity of infectious SARS-CoV-2 B.1.1.7 and B.1.351 variants to neutralizing antibodies. *Nat Med* 2021; published online March 26. DOI:](http://paperpile.com/b/rrZX91/AMpwd)[10.1038/s41591-021-01318-5](http://dx.doi.org/10.1038/s41591-021-01318-5)[.](http://paperpile.com/b/rrZX91/AMpwd)

26 [Vaidyanathan G. Coronavirus variants are spreading in India - what scientists know so far. *Nature* 2021; **593**: 321–2.](http://paperpile.com/b/rrZX91/6yydC)

27 [Liu C, Ginn HM, Dejnirattisai W, *et al.* Reduced neutralization of SARS-CoV-2 B.1.617 by vaccine and convalescent serum. *Cell* 2021; published online June 17. DOI:](http://paperpile.com/b/rrZX91/GNmG7)[10.1016/j.cell.2021.06.020](http://dx.doi.org/10.1016/j.cell.2021.06.020)[.](http://paperpile.com/b/rrZX91/GNmG7)

28 [Shoukat A, Wells CR, Langley JM, Singer BH, Galvani AP, Moghadas SM. Projecting demand for critical care beds during COVID-19 outbreaks in Canada. *CMAJ* 2020; **192**: E489–96.](http://paperpile.com/b/rrZX91/jVZ3r)

29 [Moghadas SM, Shoukat A, Fitzpatrick MC, *et al.* Projecting hospital utilization during the COVID-19 outbreaks in the United States. *Proc Natl Acad Sci U S A* 2020; **117**: 9122–6.](http://paperpile.com/b/rrZX91/IVVyo)

30 [Garg S, Kim L, Whitaker M, *et al.* Hospitalization rates and characteristics of patients hospitalized with laboratory-confirmed Coronavirus disease 2019 - COVID-NET, 14 states, March 1-30, 2020. *MMWR Morb Mortal Wkly Rep* 2020; **69**: 458–64.](http://paperpile.com/b/rrZX91/ZRsJm)

31 [Team CC-19 R, CDC COVID-19 Response Team, Chow N, *et al.* Preliminary Estimates of the Prevalence of Selected Underlying Health Conditions Among Patients with Coronavirus Disease 2019 — United States, February 12–March 28, 2020. MMWR. Morbidity and Mortality Weekly Report. 2020; **69**: 382–6.](http://paperpile.com/b/rrZX91/d04oO)

32 [Nyberg T, Twohig KA, Harris RJ, *et al.* Risk of hospital admission for patients with SARS-CoV-2 variant B.1.1.7: cohort analysis. *BMJ* 2021; **373**: n1412.](http://paperpile.com/b/rrZX91/v3zl8)

33 [Twohig KA, Nyberg T, Zaidi A, *et al.* Hospital admission and emergency care attendance risk for SARS-CoV-2 delta (B.1.617.2) compared with alpha (B.1.1.7) variants of concern: a cohort study. *Lancet Infect Dis* 2021; published online Aug 27. DOI:](http://paperpile.com/b/rrZX91/RvKRP)[10.1016/S1473-3099(21)00475-8](http://dx.doi.org/10.1016/S1473-3099(21)00475-8)[.](http://paperpile.com/b/rrZX91/RvKRP)

34 [Guan W-J, Ni Z-Y, Hu Y, *et al.* Clinical Characteristics of Coronavirus Disease 2019 in China. *N Engl J Med* 2020; **382**: 1708–20.](http://paperpile.com/b/rrZX91/V1LbM)

35 [Sanche S, Lin YT, Xu C, Romero-Severson E, Hengartner N, Ke R. High Contagiousness and Rapid Spread of Severe Acute Respiratory Syndrome Coronavirus 2. *Emerg Infect Dis* 2020; **26**: 1470–7.](http://paperpile.com/b/rrZX91/EG0i6)

36 [U.S. Bureau of Labor Statistics. Number of hospitals and hospital employment in each state in 2019. 2020; published online April 6.](http://paperpile.com/b/rrZX91/LD8Pv) <https://www.bls.gov/opub/ted/2020/number-of-hospitals-and-hospital-employment-in-each-state-in-2019.htm> [(accessed May 2, 2021).](http://paperpile.com/b/rrZX91/LD8Pv)

37 [Committee on Equitable Allocation of Vaccine for the Novel Coronavirus, National Academy of Medicine, National Academies of Sciences, Engineering, and Medicine. Discussion draft of the preliminary framework for equitable allocation of COVID-19 vaccine. Washington, D.C.: National Academies Press, 2020.](http://paperpile.com/b/rrZX91/8kRYR)

38 [CDC. How CDC is making COVID-19 vaccine recommendations. 2021; published online April 29.](http://paperpile.com/b/rrZX91/et2ZS) <https://www.cdc.gov/coronavirus/2019-ncov/vaccines/recommendations-process.html?CDC_AA_refVal=https%3A%2F%2Fwww.cdc.gov%2Fcoronavirus%2F2019-ncov%2Fvaccines%2Frecommendations.html> [(accessed May 2, 2021).](http://paperpile.com/b/rrZX91/et2ZS)

39 [Centers for Disease Control and Prevention. COVID-19 vaccination demographics in the United States, National. 2021; published online May 24.](http://paperpile.com/b/rrZX91/RJgvO) <https://data.cdc.gov/Vaccinations/COVID-19-Vaccination-Demographics-in-the-United-St/km4m-vcsb> [(accessed Aug 9, 2021).](http://paperpile.com/b/rrZX91/RJgvO)

40 [Centers for Disease Control and Prevention. COVID-19 vaccination Trends in the United States, National and jurisdictional. 2021; published online May 24.](http://paperpile.com/b/rrZX91/dgtXF) <https://data.cdc.gov/Vaccinations/COVID-19-Vaccination-Trends-in-the-United-States-N/rh2h-3yt2> [(accessed Aug 9, 2021).](http://paperpile.com/b/rrZX91/dgtXF)

41 [CDC. COVID-19 Vaccines for Children and Teens. 2021; published online Nov 4.](http://paperpile.com/b/rrZX91/sQ1n) <https://www.cdc.gov/coronavirus/2019-ncov/vaccines/recommendations/children-teens.html> [(accessed Nov 12, 2021).](http://paperpile.com/b/rrZX91/sQ1n)

42 [Anderson EJ, Rouphael NG, Widge AT, *et al.* Safety and Immunogenicity of SARS-CoV-2 mRNA-1273 Vaccine in Older Adults. *N Engl J Med* 2020; **383**: 2427–38.](http://paperpile.com/b/rrZX91/XVlPT)

43 [Polack FP, Thomas SJ, Kitchin N, *et al.* Safety and Efficacy of the BNT162b2 mRNA Covid-19 Vaccine. *N Engl J Med* 2020; **383**: 2603–15.](http://paperpile.com/b/rrZX91/gWdhH)

44 [Moghadas SM, Vilches TN, Zhang K, *et al.* Evaluation of COVID-19 vaccination strategies with a delayed second dose. *PLoS Biol* 2021; **19**: e3001211.](http://paperpile.com/b/rrZX91/dJ8bP)

45 [Sah P, Vilches TN, Moghadas SM, *et al.* Accelerated vaccine rollout is imperative to mitigate highly transmissible COVID-19 variants. *EClinicalMedicine* 2021; **35**. DOI:](http://paperpile.com/b/rrZX91/vp9BL)[10.1016/j.eclinm.2021.100865](http://dx.doi.org/10.1016/j.eclinm.2021.100865)[.](http://paperpile.com/b/rrZX91/vp9BL)

46 [Pawlowski C, Lenehan P, Puranik A, *et al.* FDA-authorized mRNA COVID-19 vaccines are effective per real-world evidence synthesized across a multi-state health system. *Med (N Y)* 2021; published online June 29. DOI:](http://paperpile.com/b/rrZX91/z9xUy)[10.1016/j.medj.2021.06.007](http://dx.doi.org/10.1016/j.medj.2021.06.007)[.](http://paperpile.com/b/rrZX91/z9xUy)

47 [Abu-Raddad LJ, Chemaitelly H, Butt AA, National Study Group for COVID-19 Vaccination. Effectiveness of the BNT162b2 Covid-19 Vaccine against the B.1.1.7 and B.1.351 Variants. *N Engl J Med* 2021; published online May 5. DOI:](http://paperpile.com/b/rrZX91/qStuy)[10.1056/NEJMc2104974](http://dx.doi.org/10.1056/NEJMc2104974)[.](http://paperpile.com/b/rrZX91/qStuy)

48 [Dagan N, Barda N, Kepten E, *et al.* BNT162b2 mRNA Covid-19 Vaccine in a Nationwide Mass Vaccination Setting. *N Engl J Med* 2021; **384**: 1412–23.](http://paperpile.com/b/rrZX91/YxwyK)

49 [U.S. Food and Drug Administration. Vaccines and related biological products advisory committee December 10, 2020 meeting briefing document. U.S. Food and Drug Administration, 2020](http://paperpile.com/b/rrZX91/Y2mrc) <https://www.fda.gov/media/144246/> [(accessed July 2, 2021).](http://paperpile.com/b/rrZX91/Y2mrc)

50 [Lipsitch M, Kahn R. Interpreting vaccine efficacy trial results for infection and transmission. *Vaccine* 2021; published online June 12. DOI:](http://paperpile.com/b/rrZX91/4hevB)[10.1016/j.vaccine.2021.06.011](http://dx.doi.org/10.1016/j.vaccine.2021.06.011)[.](http://paperpile.com/b/rrZX91/4hevB)

51 [Chodick G, Tene L, Patalon T, *et al.* Assessment of Effectiveness of 1 Dose of BNT162b2 Vaccine for SARS-CoV-2 Infection 13 to 24 Days After Immunization. *JAMA Netw Open* 2021; **4**: e2115985.](http://paperpile.com/b/rrZX91/r6n0k)

52 [Vizient, Inc. COVID-19 vaccine candidates. 2021](http://paperpile.com/b/rrZX91/2i98o) <https://www.vizientinc.com/-/media/documents/sitecorepublishingdocuments/public/covid19_sidebyside_vaccinecompare.pdf>[.](http://paperpile.com/b/rrZX91/2i98o)

53 [Lopez Bernal J, Andrews N, Gower C, *et al.* Effectiveness of Covid-19 Vaccines against the B.1.617.2 (Delta) Variant. *N Engl J Med* 2021; **385**: 585–94.](http://paperpile.com/b/rrZX91/exKSj)

54 [Pouwels KB, Pritchard E, Matthews P, *et al.* Impact of Delta on viral burden and vaccine effectiveness against new SARS-CoV-2 infections in the UK. bioRxiv. 2021; published online Aug 24. DOI:](http://paperpile.com/b/rrZX91/JorjV)[10.1101/2021.08.18.21262237](http://dx.doi.org/10.1101/2021.08.18.21262237)[.](http://paperpile.com/b/rrZX91/JorjV)

55 [Bernal JL, Andrews N, Gower C, *et al.* Effectiveness of COVID-19 vaccines against the B.1.617.2 variant. bioRxiv. 2021; published online May 24. DOI:](http://paperpile.com/b/rrZX91/4TjXL)[10.1101/2021.05.22.21257658](http://dx.doi.org/10.1101/2021.05.22.21257658)[.](http://paperpile.com/b/rrZX91/4TjXL)

56 [Grannis SJ, Rowley EA, Ong TC, *et al.* Interim Estimates of COVID-19 Vaccine Effectiveness Against COVID-19-Associated Emergency Department or Urgent Care Clinic Encounters and Hospitalizations Among Adults During SARS-CoV-2 B.1.617.2 (Delta) Variant Predominance - Nine States, June-August 2021. *MMWR Morb Mortal Wkly Rep* 2021; **70**: 1291–3.](http://paperpile.com/b/rrZX91/FM7HK)

57 [Tada T, Zhou H, Samanovic MI, *et al.* Comparison of Neutralizing Antibody Titers Elicited by mRNA and Adenoviral Vector Vaccine against SARS-CoV-2 Variants. *bioRxiv* 2021; published online Aug 6. DOI:](http://paperpile.com/b/rrZX91/vmMM)[10.1101/2021.07.19.452771](http://dx.doi.org/10.1101/2021.07.19.452771)[.](http://paperpile.com/b/rrZX91/vmMM)

58 [Sadoff J, Gray G, Vandebosch A, *et al.* Safety and Efficacy of Single-Dose Ad26.COV2.S Vaccine against Covid-19. *N Engl J Med* 2021; **384**: 2187–201.](http://paperpile.com/b/rrZX91/qZtq)

59 [IISInfo. COVID-19 vaccination demographics in the United States,national. 2021; published online May 24.](http://paperpile.com/b/rrZX91/FeBG) <https://data.cdc.gov/Vaccinations/COVID-19-Vaccination-Demographics-in-the-United-St/km4m-vcsb> [(accessed Nov 16, 2021).](http://paperpile.com/b/rrZX91/FeBG)

60 [Bajema KL, Wiegand RE, Cuffe K, *et al.* Estimated SARS-CoV-2 Seroprevalence in the US as of September 2020. *JAMA Intern Med* 2021; **181**: 450–60.](http://paperpile.com/b/rrZX91/LMiif)
